# Meristem genes are essential for the vegetative reproduction of *Kalanchoë pinnata*

**DOI:** 10.3389/fpls.2023.1157619

**Published:** 2023-05-08

**Authors:** Francisco Jácome-Blásquez, Minsung Kim

**Affiliations:** School of Biological Sciences, Faculty of Biology, Medicine and Health, The University of Manchester, Manchester, United Kingdom

**Keywords:** asexual and vegetative reproduction, leaf crenulations, plantlet formation, *SHOOTMERISTEMLESS* (STM), *WUSCHEL*, stem cells, meristem, *Kalanchoë* species

## Abstract

Several *Kalanchoë* species reproduce asexually by forming plantlets in the leaf crenulations. Some species produce plantlets incessantly *via* somatic embryogenesis and organogenesis, whereas others exclusively develop plantlets after leaf detachment, presumably through organogenesis. *SHOOT MERISTEMLESS* (*STM*), which mediates SAM functions, appears to be involved in *Kalanchoë* plantlet formation, suggesting that meristem genes may be essential for plantlet formation. However, the genetic regulatory network for establishing and maintaining plantlet primordia in *Kalanchoë* remains elusive. Here, we showed that meristem genes were differentially expressed in the leaf crenulations of *K. pinnata* during plantlet development after leaf detachment. The regulatory interactions among these meristem genes are largely conserved in *K. pinnata* crenulations. Moreover, transgenic antisense (AS) plants with lower expression of these key meristem genes formed significantly fewer plantlets with some morphological defects, suggesting that the meristem genes play an important role in plantlet formation and development. Our research revealed that key meristem genetic pathways were co-opted to the leaf margin to facilitate the unique asexual reproduction mechanism in *K. pinnata*. This also highlights how evolutionary tinkering invents new structures such as epiphyllous buds and plantlets by rewiring pre-existing genetic pathways.

## 1 Introduction

Plants maintain a source of undifferentiated stem cells in the shoot apical meristem (SAM) and root apical meristem (RAM), which differentiate for growth and tissue self-renewal (Sablowski, 2004; Zhu, 2017). Plants have also secondary stem cell niches, such as axillary buds and epiphyllous buds (EBs), which are established post-embryonically in the existing organs, remain dormant and under stress or other external conditions acquire SAM or RAM functions (Dinneny and Benfey, 2008; Ten Hove et al., 2015). Environmental cues, positional cues and phytohormones influence the fate of stem cells (Gaillochet and Lohmann, 2015; Sengupta et al., 2018). The establishment and maintenance of stem cells in secondary niches involve the reacquisition of stem cell identity by a group of cells, followed by morphogenetic processes. Transcriptional regulators, hormones and mobile signals from neighbouring cells can revert differentiation and maintain stem cell identity (Gaillochet and Lohmann, 2015; Galli and Gallavotti, 2016). Previous studies have reported the presence of undifferentiated cells within the parenchyma of the leaves of most succulent plants, which enabled the regeneration of new plants from leaf-cuttings (Gorelick, 2015).

The SAM activity requires a balance between the number of cells recruited for organ formation and the cells to self-sustain (Heidstra and Sabatini, 2014). The main regulatory mechanism in the SAM to preserve stem cells includes the *WUSCHEL* (*WUS*) and *CLAVATA* (*CLV*) gene negative feedback loop (Schoof et al., 2000; Somssich et al., 2016; Fletcher, 2018). In *Arabidopsis thaliana*, *WUS* is required for the maintenance of undifferentiated cells in the SAM (Mayer et al., 1998; Bäurle and Laux, 2005). *WUS* is expressed in the organising centre (OC) and triggers the expression of *CLV3* in the central zone (CZ), which simultaneously restricts *WUS* to the OC (Bäurle and Laux, 2005). CLV3 binds to CLV1 leucine-rich repeat-receptor-like kinase (LRR-RLK) heterodimers with CLV2 LRRreceptor-like protein (RLP) and its co-receptor CORYNE (CRN) protein to inhibit *WUS* expression (Fletcher, 2018). It has been shown that CLV3 also binds to CLV2-CRN to inhibit *WUS* independently from *CLV1* (Bleckmann et al., 2009; Guo et al., 2010). This *CLV*/*WUS* negative feedback loop determines the area of the *WUS* domain, which appears to be a well-conserved mechanism within the plant kingdom (Bäurle and Laux, 2005; Somssich et al., 2016). In addition, *Arabidopsis HAIRY MERISTEM1* (*HAM1*) and *HAM2* genes are conserved cofactors of WUS/WUSCHEL-LIKE HOMEOBOX (WOX) proteins, which are also essential for SAM maintenance (Zhou et al., 2015). *HAM1* and *HAM2* are co-expressed with *WUS* in the CZ, however, it has been shown that *WUS* can only activate *CLV3* in the absence of HAM proteins, avoiding *WUS* repression in the CZ by CLV3 (Yadav et al., 2011). LRR-RLK *BARELY ANY MERISTEM 1* (*BAM1*) sharing 81% similarity to *CLV1* (DeYoung et al., 2006), functions analogously to *CLV1*, binding directly to CLV3. Double knock-out of *CLV1* and *BAM1* showed stem cell overproliferation, similar to the phenotypes observed in *CLV3* loss-of-function (Shinohara and Matsubayashi, 2015).

The ectopic expression of *WUS* induced the formation of stem-like cells and somatic embryos in *Arabidopsis* (Schoof et al., 2000). A SAM-like function can be also acquired by the ectopic expression of *WUS* in the leaves, triggered by the presence of cytokinins (Gordon et al., 2009). *wus* mutants showed termination of the meristematic activity after forming a few organs due to the lack of stem cell renewal (Endrizzi et al., 1996). In contrast, *clv* mutants displayed stem cell over-proliferation and disorganised stem cell arrangement (Brand, 2000; Schoof et al., 2000). It has been shown that the external application of cytokinins significantly downregulates *CLV1* expression, resulting in upregulated *WUS* expression, suggesting that *WUS* might be activated by cytokinins (Lindsay et al., 2006). However, cytokinins application to *clv3* and *clv1* loss-of-function mutants in *Arabidopsis* induced *WUS* expression, suggesting a *CLV-*independent mechanism of *WUS* regulation (Gordon et al., 2009).

The *KNOX* homeodomain *SHOOT MERISTEMLESS* (*STM*) gene is essential for SAM maintenance along with *WUS* (Clark et al., 1996; Long et al., 1996; Lenhard et al., 2002). *STM* knocked-out phenotypes completely abolished SAM formation in *Arabidopsis* (Long et al., 1996; Aida et al., 1999; Scofield et al., 2014), whereas mild *STM* downregulation perturbed the SAM organisation and compromised its post-embryonic maintenance (Endrizzi et al., 1996; Long et al., 1996). *WUS* and *STM* ectopically expressed in the leaves of *Arabidopsis* triggered a robust subset of meristem functions, including the WUS/CLV pathway, and thus organogenesis (Gallois et al., 2002). Alternatively, expression analyses have reported that *CUP SHAPED COTYLEDON1* and *2* (*CUC1* and *2*) are required for *STM* expression during *Arabidopsis* embryogenesis (Aida et al., 1999). Loss of function mutants of *STM* and *CUC* genes share similar phenotypes with fused cotyledons (Endrizzi et al., 1996; Takada et al., 2001; Scofield et al., 2018). *cuc1* and *2* double mutants were unable to develop an embryonic SAM, as *STM* was not activated (Aida et al., 1999; Takada et al., 2001). Postembryonically, *CUC2* interacts with *PINFORMED1* (*PIN1*) and miR164 to form serrations in the leaves of *Arabidopsis* (Nikovics et al., 2006; Bilsborough et al., 2011).

The genus *Kalanchoë* comprises several species that reproduce vegetatively by forming plantlets in the leaf margins. While some species such as *K. pinnata* and *K. prolifera* only form plantlets after leaf excision (induced plantlet-forming species), other *Kalanchoë* species such as *K. daigremontiana* and *K. laetivirens* form plantlets incessantly from pedestal-like structures located in the leaf serrations (Garces et al., 2007). While *K. pinnata* can reproduce sexually, under stress conditions, *K. pinnata* reproduces through the formation of plantlets from bud-like structures, known as epiphyllous buds (EBs) on leaves crenulations (Sawhney et al., 1994; Kulka, 2008; Laura et al., 2013). *K. pinnata* EBs on the leaf crenulations stay dormant until leaf detachment or severe damage which triggers a dormancy-release mechanism forming plantlets through organogenesis (Garces et al., 2007). Maintaining cell pluripotency on the leaves appears to be the basis for this mode of vegetative reproduction (Zhu, 2017), capable of forming new plants. It is speculated that *K. pinnata* EBs are organised during leaf formation (Naylor, 1932) and remain latent until the leaf is detached from the mother plant (Sawhney et al., 1994; Rajsekhar et al., 2016). Plantlet initiation in inducible plantlet-forming *Kalanchoë* species is triggered by hormonal influence (Sawhney et al., 1994). In *K. marnierianum*, exogenous cytokinin applications promoted dormancy in the EBs, inhibiting plantlet formation after leaves were detached (Kulka, 2006). Moreover, the auxin transport inhibitor, 2, 3, 5-triiodobenzoic acid (TIBA) restricted the formation of roots in plantlets of *K. marnierianum*, suggesting that the root formation depends on auxin signals from the plantlet SAM (Kulka, 2008).

Molecular studies in *Kalanchoë* have confirmed that *STM* expression in the leaves is only present in the species that form plantlets on the leaf margins (Garces et al., 2007). *STM* knockout abolished plantlet formation in *K. daigremontiana* (Garces et al., 2007). This might suggest that *STM* establishes meristematic competency in the leaf crenulations of *Kalanchoë* enabling the plantlet formation. Incessant (constitutive) plantlet-forming species such as *K. daigremontiana* also ectopically expressed embryo genes, *FUSCA3* (*FUS3*) and an unfunctional mutated version of *LEAFY COTYLEDON1* (*LEC1*), in leaves, which allowed the formation of leaf embryos and subsequently bypass dormancy (Garces et al., 2007). The expression of a functional *AtLEC1* in *K. daigremontiana* severely disrupted the process of plantlet formation, inducing dormancy in foliar embryos and the accumulation of seed-specific oils in the leaf margins (Garces et al., 2014). *LEC1* and *LEC2* in *Arabidopsis* induce the accumulation of seed-specific oils to prevent the zygotic embryo from desiccation and are a major C source for germinating seedling (Pelletier et al., 2017). During *Arabidopsis* embryogenesis, *FUS3* works as a transcriptional activator facilitating embryo development and avoiding early seedling transition (Wang and Perry, 2013). *FUS3* and *LEC2* form a complex, which binds to the auxin biosynthesis gene, *YUCCA4* (*YUC4*), inducing lateral root development in *Arabidopsis* (Tang et al., 2017).

Here we showed that key meristem genes such as *K. pinnata WUS* (*KpWUS*) and *K. pinnata STM* (*KpSTM*) play important roles in the organisation and maintenance of stem cells during plantlet formation in an induced plantlet-forming species *K. pinnata*. We also showed that *K. pinnata CLV2* (*KpCLV2*) delimited *KpWUS* expression in the epiphyllous buds, and *K. pinnata CUC2* (*KpCUC2*) modulated the *STM* expression and its downregulation affected the indentation depth in the leaves of *K. pinnata*, disturbing the formation of EBs. This work highlights the acquisition of meristem competency and key meristem pathways as a key factor to enable vegetative reproduction in the leaves of *K. pinnata*.

## 2 Materials and methods

### 2.1 Plant materials and growth conditions

*Kalanchoë pinnata* wild-type (WT) and antisense (AS) transgenic plants were potted into Levington’s F2 compost (Scott’s Miracle-Gro, UK), perlite (Sinclair Horticulture, Ltd, UK), and Vermiculite (Sinclair Horticulture, Ltd, UK) mix in a 6:1:1 ratio. The plants were grown in a Percival Scientific growth chamber AR-60L at 23°C, illuminated with fluorescent lights in a short-day condition photoperiod 8 hours/16 hours. 1 cm^2^ explants were dissected from young fully-grown whole leaves and used for plant transformation. For scanning electron microscopy (SEM), toluidine blue staining (TB), immunolocalisation and RT-qPCR, tissues from margins of smaller than 0.5 cm leaves were used.

### 2.2 RNA extraction for Illumina 2000 sequencing analysis

Total RNA was extracted from 0.3 cm^2^ tissues at the leaf notches from 3 biological replicates of *K. pinnata* wild type at different time points after leaf detachment (0, 4, 24 and 48 hours). 0 hours after leaf detachment was used as a control in addition to tissue samples obtained from the mid-section margin of the leaves 48 hours after leaf detachment, where no plantlet formation may occur. RNA extraction and sequencing analysis were performed as described by (Jácome-Blásquez et al. (2022).

### 2.3 Gene cloning and vector assembly

*KpWUS*, *KpCLV2*, *KpSTM*, and *KpCUC2* clones were isolated using gene-specific primers based on *WUS*, *CLV2*, *STM* and *CUC2* orthologs from *K. laxiflora* and *K. fedtschenkoi* sequences (found on Phytozome v12.1, JGI, University of California); **Supplementary Table S1**. A 455 bp fragment of *KpWUS* located in exon 1 (Accession OQ674700), a 942 bp fragment of *KpCLV2* in exon 2 (Accession OQ621804), a 258 bp fragment of *KpCUC2* in exon 1 (Accession OQ621805) and a 333 bp fragment of *KpSTM* in exon 4 (Accession OQ674701) were cloned to make AS constructs. The sequences were amplified and cloned using Q5^®^ High-Fidelity DNA Polymerase (New England Biolabs, USA). The PCR products were cleaned up (Nucleospin^®^ gel and PCR Clean-Up Kit; Macherey-Nagel, Germany) and then ligated into pGEM^®^-T Easy (Promega, USA). The fragments were ligated using Golden Gate assembly in AS orientation with the cauliflower mosaic virus (CaMV) *35S* Promoter and Terminator for *KpWUS* and *KpCLV2* AS constructs, and CaMV *35S* Promoter and the *Nopaline Synthase* (*NOS*) Terminator for *KpCUC2* and *KpSTM* AS constructs. The constructs were then inserted into a modified *pBI121* vector. Subsequently, the constructs were transformed into *Escherichia coli* strain *DH5α* for selection. Correct constructs were then transformed into *Agrobacterium tumefaciens* strain *LBA4404* by electroporation and confirmed with culture PCR.

### 2.4 *K. pinnata* transformation

WT *K. pinnata* plants were transformed with *35S::KpWUS*, *35S::KpCLV2*, *35S::KpCUC2* and *35S::KpSTM* AS as previously described (Garcěs and Sinha, 2009). Transformed *Agrobacterium* (LB4404 strain) was grown for 48 hours in Luria Bertani (LB) medium without NaCl supplemented with 50 mg/L Rifampicin, 100 mg/L Kanamycin and 100 mg/L Streptomycin on an orbital shaker, at 30°C and 250 rpm in the dark. OD_600_ was confirmed until 0.5 o.u. was achieved. The cells were centrifuged at 5500 RFC for 15 minutes and resuspended in 0.5 Murashige and Skoog (MS) medium supplemented with 100 μm of acetosyringone. 1 cm^2^ leaf fragments previously disinfected with absolute ethanol and commercial bleach (13%) were co-cultured for 2 hours in an orbital shaker in the dark. Inoculated leaf tissues were cultured in the dark in MS medium without the addition of antibiotics for two days, afterwards transferred to SIM (shoot-inducing media) supplemented with 100 mg/L TDZ and 10 mg/L IAA and antibiotics 40 mg/L Kanamycin and 500 mg/L Carbenicillin. After two weeks, these were transferred to SIM with 100 mg/L Kanamycin and 500 mg/L Carbenicillin. We did subculture every 15 days for four months. Once leaves were formed, the explants were transferred to RIM (root-inducing media) containing 0.5 MS media supplemented with 30 g/L sucrose, 7.5 g/L agar, and 5.8 pH adjusted. Roots formed after three weeks, and then the plants were established *ex vitro* plants into a mix of Levington’s F2 compost (Scott’s Miracle-Gro, UK), perlite (Sinclair Horticulture, Ltd, UK), and Vermiculite (Sinclair Horticulture, Ltd, UK) in a 6:1:1 ratio.

### 2.5 Genotyping AS lines

Quick DNA prep for PCR protocol was performed to obtain DNA (Weigel and Glazebrook, 2002). PCR was implemented with Q5^®^ High-Fidelity DNA polymerase and BioTaq™ polymerase (Bioline, UK). The primers used were *KpWUS*, *KpCLV2*, *KpCUC2, KpSTM* and *M13* (**Supplementary Table S2**). Successful incorporation of the insert was also confirmed detecting *NTPII* forward and reverse primers. PCR settings were the ones recommended in the Q5^®^ protocol, with an annealing temperature of 56°C and an extension time of 30 seconds for 35 cycles.

### 2.6 Phenotyping AS lines

Leaves were excised from the mother plant, arranged on a dry white paper sheet, and kept in a growth chamber. New plantlet regeneration from the leaf primordia was scored every 3 days up to 21 days for WT and AS lines. New plantlets were scored when they became visible, approximately 0.5 mm in length. A total of 25 leaves from 3 - 5 independent AS lines (several leaves from the same plant) were used for this experiment. Plantlet emergence was scored with Microsoft Excel version 16.16.27, and GraphPad Prism 9.2.0 was used to plot the graphs and perform the ANOVA analysis with Dunnett’s Multiple Comparisons.

### 2.7 Scanning electron microscopy

Scanning electron microscopy (SEM) was performed on leaf crenulations. Fresh tissue was fixed according to the previous protocol (H. Garces & Sinha, 2009). Samples were examined using a Quanta 650 FEG ESEM with an Energy-Dispersive Spectroscopy (EDS) Bruker XFlash^®^ 6/30 SDD by FEI/ThermoFisher SEM.

### 2.8 Quantitative reverse transcription polymerase chain reaction (RT-qPCR)

EBs of WT *K. pinnata* and individual *KpWUS*, *KpCLV2*, *KpSTM* and *KpCUC2* AS lines were excised and frozen in liquid nitrogen. Total RNA was extracted using the RNeasy Plant Mini Kit (Qiagen, USA) according to a modification (Gehrig et al., 2000; Garces and Sinha, 2009a). RNA was treated with RQ1 DNase (Promega, USA) and cDNA synthesis was achieved at 45°C for 1 hour with the Tetro cDNA Synthesis kit (Bioline, UK). The RT-qPCR reaction was prepared using 100 ng of cDNA in triplicate of three biological replicates in a 20 μL reaction containing 10 μL of SensiFAST™ SYBR Hi ROX kit (Bioline, UK) and 1 mM of each primer. Primer sequences are shown in **Supplementary Table S2**. The reaction was performed in a StepOnePlus™ Real-Time PCR machine with StepOne™ software v2.3. *18S Ribosomal RNA* gene was used as a control gene with an annealing temperature of 60°C. The data analysis was done using the Comparative CT method. Microsoft Excel version 16.16.27 was used for data processing and GraphPad Prism 9.2.0 to plot the graphs and the ANOVA analysis with Dunnett’s Multiple Comparisons using WT as the control.

### 2.9 Immunolocalisation

Tissue samples from *K. pinnata* epiphyllous buds were fixed and sectioned for immunolocalisation according to the previous protocol (Garces and Sinha, 2009b). Following the sectioning on slides, paraplast wax (Sigma, USA) was removed with a 100% histological clearing agent (Histoclear II) (National diagnostics, USA). Sections were rehydrated in ethanol series with a final blocking incubation in phosphate-buffered saline (PBS) buffer with 1% bovine serum albumin (BSA). The slides were incubated with a specific WUS antibody previously examined in *K. daigremontiana* (Santa Cruz Biotechnology, catalogue number-sc12587, USA; 1:500 dilution) in 1xPBS with 1% BSA for 2 hours at 4°C. After three washes in 1xPBS, slides were incubated with AP-conjugated Donkey Anti-Goat IgG secondary antibody (Promega, USA; 1:400 dilution). The samples were developed in the dark for 35 minutes for colourimetric detection. The slides were washed in 1xPBS and mounted with DPX Mountant for Histology (Sigma-Aldrich, USA). The slides were observed and photographed in a compound microscope GXML 2800 with an integrated camera GXCAM HI Chrome-SMII (GT Vision, UK).

### 2.10 Image acquisition

*K. pinnata* leaf primordia in WT and AS lines were photographed using a GXCAM-Eclipse (0654) Wi-Fi camera, attached to S8 APO Stereo Microscope (Leica, Germany). To photograph whole leaves of *K. pinnata* in WT and AS lines, a 12-megapixel Ultrawide: *f*/2.4, Wide: *f*/1.6 Telephoto: *f*/2.2 aperture phone camera was used. Immunolocalisation slides were photographed with a GXCAM HI Chrome-SMII attached to a compound microscope GXML 2800 (GT Vision, UK).

## 3 Results

### 3.1 Leaf growth, EB and plantlet formation

**Figure 1:**
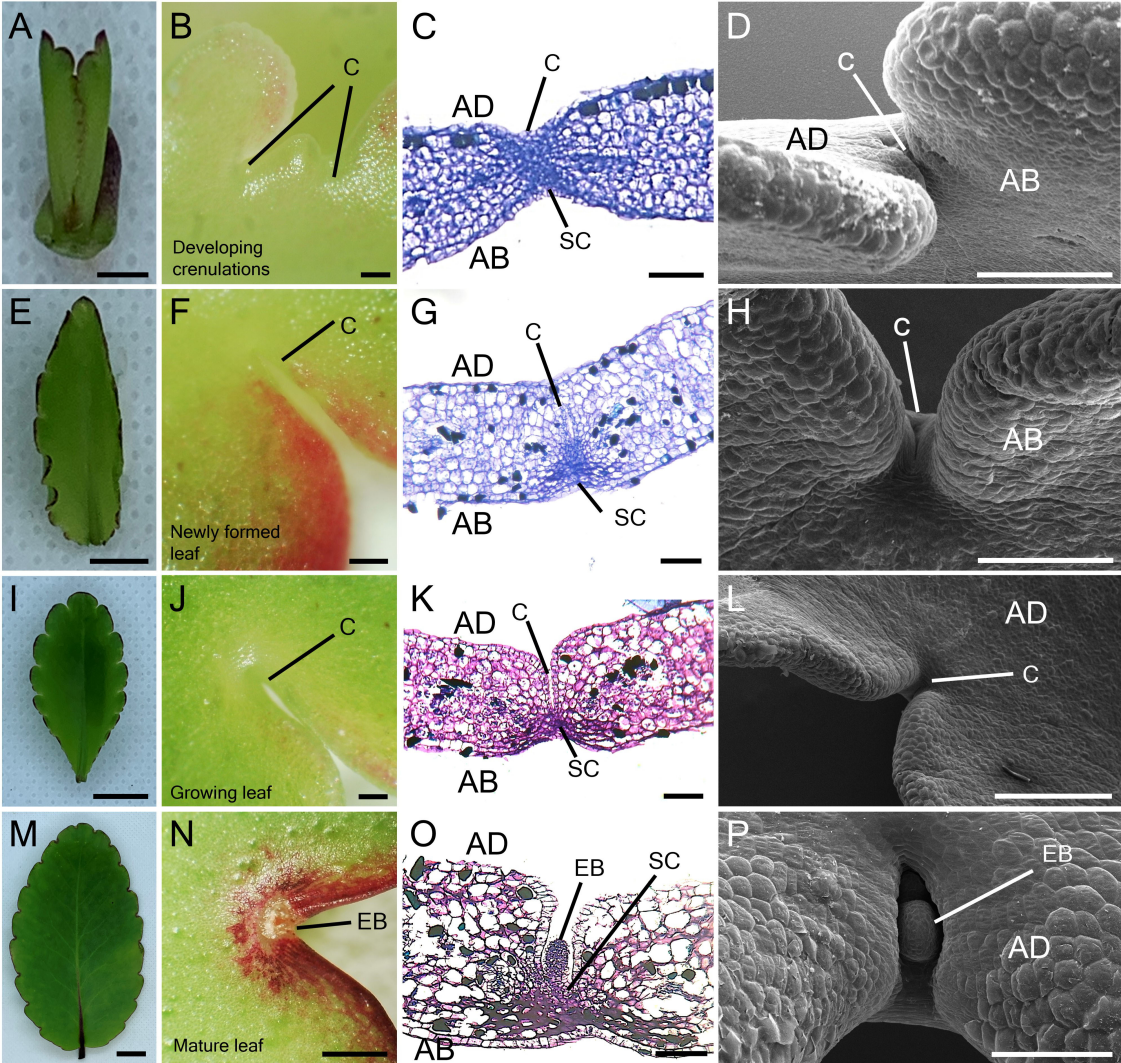
Leaf growth and epiphyllous bud formation in *K. pinnata*. Morphological appearance of the leaf growth **(A, E, I, M)**. Microscopic imaging of the adaxial side of developing leaf crenulations **(B, F, J)** and abaxial side (N). TB staining of developing leaf crenulations with small cells resembling stem cells in the crenulation area depicts the presence of stem cells which later develop into EBs **(C, G, K, O)**. SEM imaging of developing crenulations with no superficial epiphyllous bud in the crenulation area **(D, H, L)** until the leaf is fully developed **(P)**. Scale bar: 1 mm **(A, N)**; 0.5 mm **(B, F, J)**; 0.1 mm **(C, G, K)**; 500 µm **(L)** and 150 µm **(D, H, P)**. AB; abaxial side, AD; adaxial side, C; Crenulation, SC; Stem cells, EB; Epiphyllous bud.

**Figure 2:**
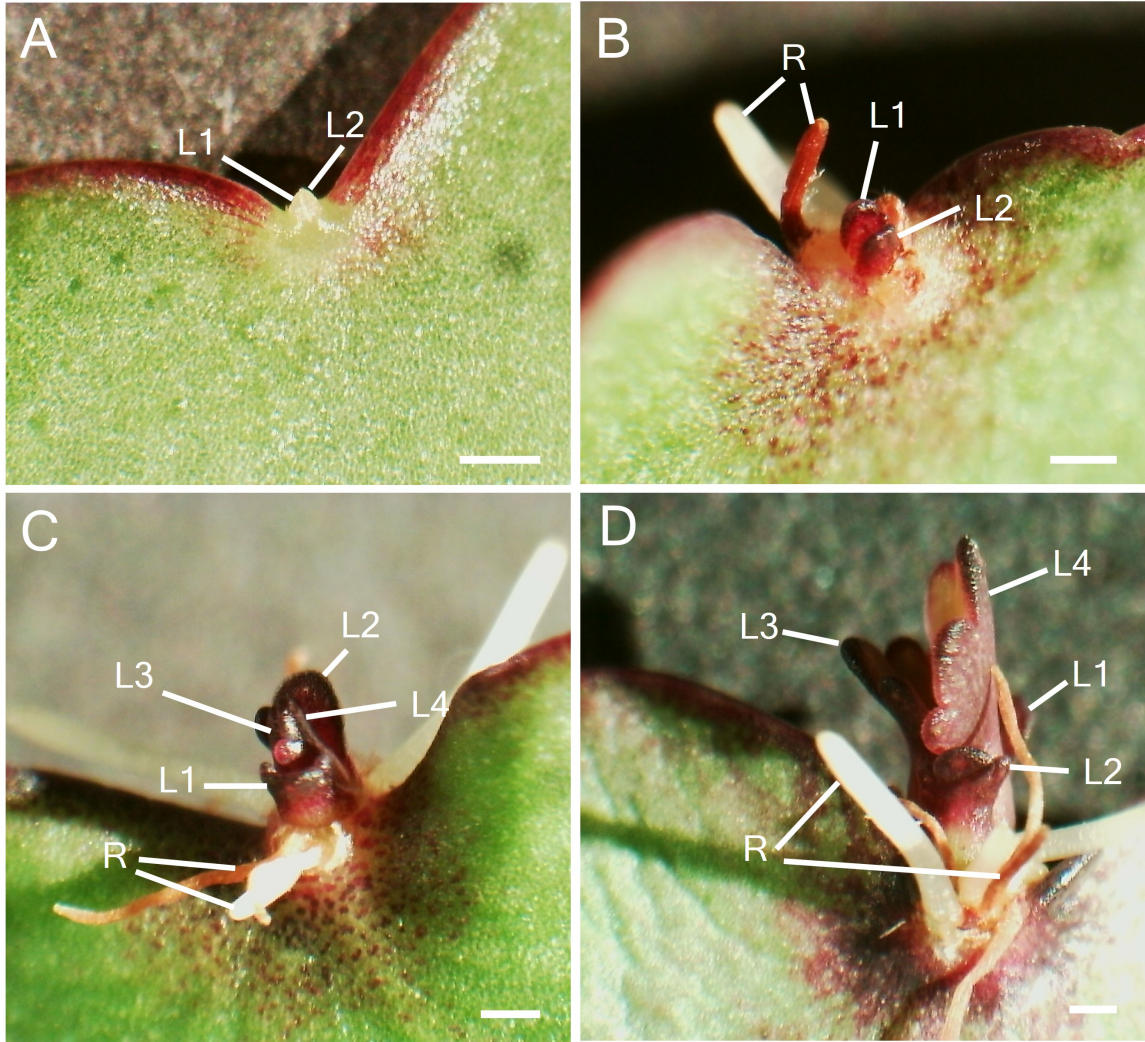
Plantlet formation after leaf detachment. **(A)**. Leaf EB after 9 days of leaf detachment with the emergence of leaves (L1 and L2). **(B)** EB after 15 days of leaf detachment with enlarged L1 and L2 and root development **(C)** Emergence of second pair of leaves (L3 and L4) and root growth over time. **(D)** Growth and development of leaves and roots. Scale bar: 1 mm. L1; Leaf 1, L2; Leaf 2, L3; Leaf 3, L4; Leaf 4, R; root.

To study the participation of meristem genes in EBs and subsequently plantlet formation in *K. pinnata* leaves, we initially examined the leaf crenulations during leaf development. A newly emerged leaf of a few millimetres long started forming crenulations in the distal region (**Figure 1A**) without any visible epiphyllous buds (**Figures 1B, D**). The crenulations presented no superficial formation of the EBs, however, Toluidine Blue (TB) staining sections revealed numerous small cells resembling stem cells (SC) congregated at the crenulation areas (**Figure 1C**). The crenulations in small (< 1 cm) younger leaves were fully formed (**Figure 1E**), nevertheless, there was still no evident EBs formation (**Figures 1F, H**). The TB-stained sections showed congregated small presumptive SC around the crenulations (**Figure 1G**). Growing leaves (< 5 cm; **Figure 1I**) presented deeper crenulations. The crenulation area at this stage exhibited subtle differences in colour in comparison with the mid-sections of the leaf (**Figure 1J**). TB sections revealed deep crenulations with presumptive SC concentrated particularly in the bottom of the crenulation gorge (**Figure 1K**). However, the crenulations captured in SEM images showed no clear evidence of EBs presence (**Figure 1L**). In contrast, fully grown (> 5 cm) leaves (**Figure 1M**) exhibited EBs when observed in a dissecting microscope. At the leaf margin where plantlets are speculated to form, EB consists of a globular protuberance located in the abaxial side of the leaf crenulation (**Figure 1N**). TB-stained sections in mature leaves showed an elongated EB structure positioned in the centre of the leaf crenulation composed of small SC (**Figure 1O**). These crenulations showed a protuberance with an emerging globular-shaped EB when observed in SEM (**Figure 1P**). Following the leaf detachment, plantlet leaf primordia (L1 and L2) emerged from the EBs after approximately 9 days (**Figure 2A**). After 15 days following leaf detachment, the EB had leaf 1 (L1) enlarged, and leaf 2 (L2) and roots (R) became visible to the naked eye (**Figure 2B**). 20 days after leaf detachment, plantlets formed more leaves (L3 and L4) while L1, L2 and roots continued to develop (**Figure 2C**) and grow. **Figure 2D** illustrates a 25-day-old plantlet. The plantlets have functional leaves with crenulations and elongated roots; however, these remain attached to the excised senescent mother leaves.

### 3.2 Differentially expressed meristem genes after leaf detachment

**Figure 3:**
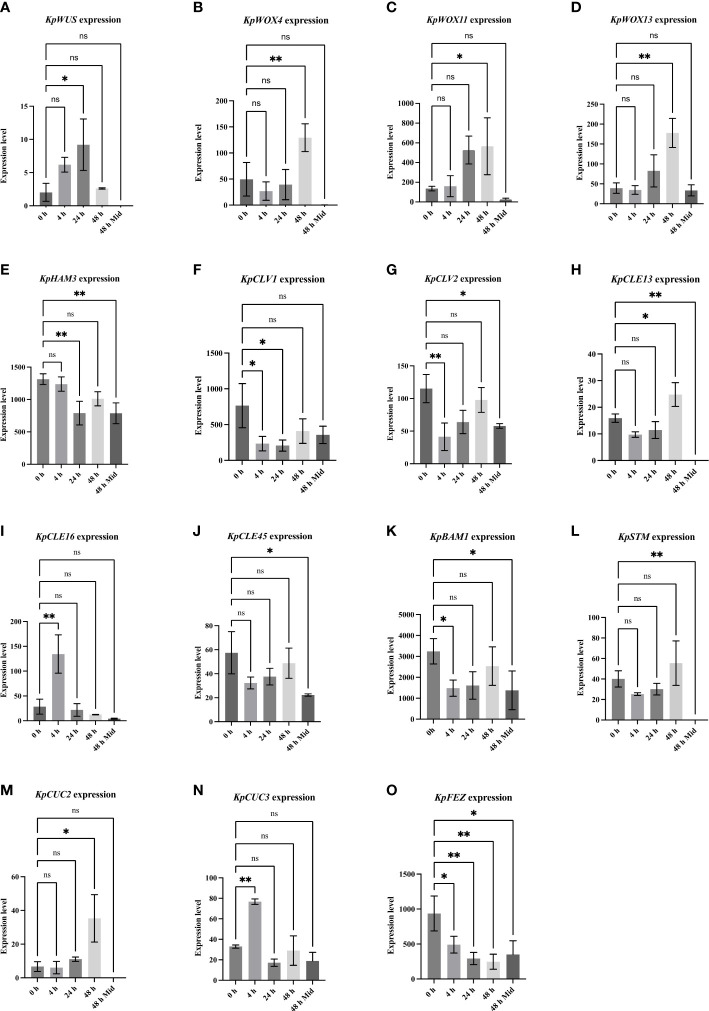
Differential expression of meristem genes at different time points (0, 4, 24, and 48 hours in leaf crenulations and 48 hours in mid-sections of the leaves) after leaf detachment. One-Way ANOVA with Dunnett’s Multiple comparisons, ns (non-significant); * (P-value <0.01); ** (P value <0.001). Errors bars represent the 95% confidence interval. The data was taken from (Jácome-Blásquez et al., 2022).

To reveal meristem genes involved in plantlet formation, data taken from a previous RNA sequencing analysis was evaluated allowing the detection of early changes in gene expression following the leaf detachment. RNA was extracted from EBs of leaves at 0, 4, 24 and 48 hours, after leaf detachment, and from mid-sections of the detached leaves after 48 hours, with presumably no plantlet formation activity (negative control) (Jácome-Blásquez et al., 2022). Several key meristem genes and closely-related genes were differentially expressed in the EBs after leaf detachment. However, differential expression patterns of these genes varied across time points. From this data, we monitored the expression of meristem genes known to be key in the formation and maintenance of the SAM in *Arabidopsis*. *KpWUS* expression was upregulated significantly 24 hours after leaf detachment (p=0.033), followed by downregulation after 48 hours. *KpWUS* expression was not detected at the mid-sections of the leaves (**Figure 3A**). *WOX* genes were also expressed in the EBs. *KpWOX4* (p=0.007), *KpWOX11* (p=0.033), and *KpWOX13* (p=0.013) showed similar expression patterns, being significantly upregulated 48 hours after the leaf detachment. *KpWOX4* was undetectable at the mid-sections of the leaves, whereas *KpWOX11* and *KpWOX13* were slightly expressed (**Figures 3B-D**). *KpHAM3* gene was present in the EBs and the mid-sections of leaves, however, its expression was higher in the EBs (p=0.002), and significantly downregulated 24 hours after leaf detachment (p=0.002; **Figure 3E**). *KpCLV1* and *KpCLV2* transcripts were detected in the EBs and downregulated following leaf detachment after 4 and 24 hours (p=0.001; **Figure 3F**) and 4 hours (**Figure 3G**), respectively. *KpCLAVATA3*/*ENDOSPERM SURROUNDING REGION-RELATED13* (*KpCLE13*) was found upregulated in the EBs 48 hours after leaf detachment (p=0.028; **Figure 3H**). *KpCLE16* expression was found first upregulated 4 hours after leaf detachment (p=0.002), then downregulated after 24 and 48 hours (**Figure 3I**). *KpCLE45* was significantly overexpressed in the EBs of attached leaves when compared to the mid-section of the leaves (p=0.032), however, its expression did not vary between the different time points after leaf detachment (**Figure 3J**). *KpBAM1* was upregulated in the EBs when compared to the mid-sections of the leaves. Its expression was downregulated 4 h after the leaf excision (p=0.044; **Figure 3K**). The *KpSTM* gene was absent at the mid-section of the leaves but present in the EBs, however, its expression continued unchanging across the different time points after leaf detachment (**Figure 3L**). *KpCUC2* and *KpCUC3* transcripts were also present in the EBs. *KpCUC2* was completely absent in the mid-sections of the leaves and highly upregulated (p=0.014) after 48 hours following the leaf detachment (**Figure 3M**). *KpCUC3* was upregulated 4 hours (p=0.009) after leaf detachment, following downregulation after 24 hours (**Figure 3N**). The *NAC* domain transcription factor ortholog, *KpFEZ* was detected upregulated in the EBs of attached leaves in comparison to the mid-section (p=0.016). *KpFEZ* showed a gradual downregulation at 4 hours (p=0.035), 24 hours (p=0.009) and 48 hours (p=0.003) after leaf detachment (**Figure 3O**).

### 3.3 Phenotyping of *KpWUS*, *KpCLV2*, *KpCUC2* and *KpSTM* AS lines

**Figure 4:**
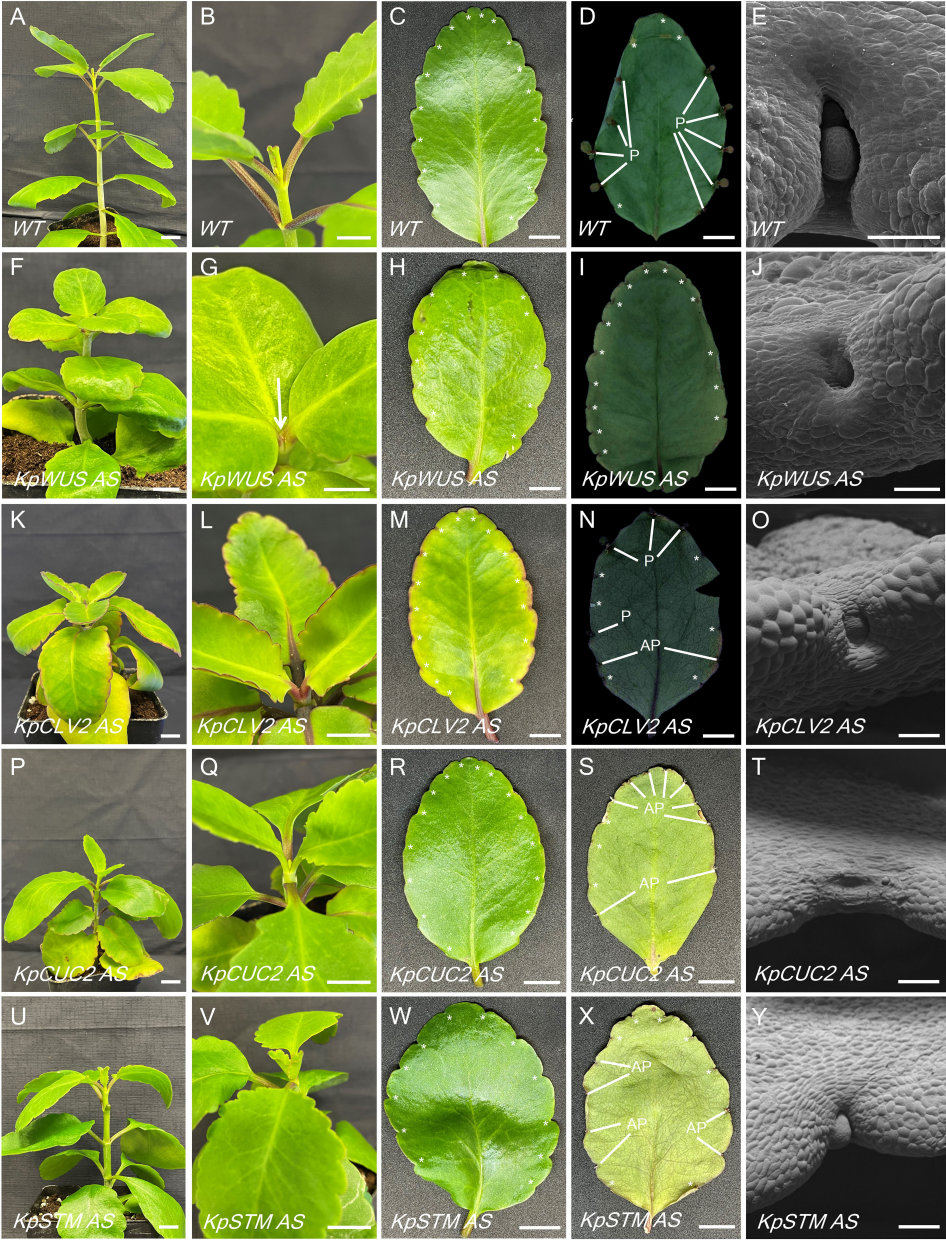
Phenotypic comparison between WT and *KpWUS*, *KpCLV2*, *KpCUC2* and *KpSTM* antisense (AS) lines. Full plant images **(A, F, K, P, U)**; newly emerged leaves in the shoot **(B, G, L, Q, V)**; individual leaves imaging at 0 hours **(C, H, M, R, W)** and 20 days **(D, I, N, S, X)** following leaf detachment, scale bars: 1 cm. SEM imaging of the leaf crenulation area **(E, J, O, T, Y)** scale bar: 250 µm. P; plantlet, AP; aborted plantlet, *; leaf crenulation.

To elucidate the role of meristem genes in plantlet formation, AS lines were generated by expressing AS fragments of *KpWUS*, *KpCLV2*, *KpCUC2* and *KpSTM* under the cauliflower mosaic virus 35S promoter. These genes were chosen as they are key players in the *STM* pathway which is involved in *K. daigremontiana* plantlet formation. The AS lines were confirmed by PCR amplifying the transgenes and the *NPTII* gene (**Supplementary Figure S1**). We confirmed multiple independent lines: three for *KpWUS* AS, eight for *KpCLV2* AS, five for *KpCUC2* AS and five for *KpSTM* AS (**Supplementary Figure S1**). *K. pinnata* WT grows vertically with a decussate leaf arrangement (**Figure 4A**). **Figure 4B** shows newly formed leaves emerging from the SAM in WT, and a new leaf pair emerged while the older pair was still growing. The mature leaves of *K. pinnata* WT have approximately 18 crenulations distributed on the margins (**Figure 4C**, asterisks). **Figure 4D** shows a leaf 20 days after detachment with plantlets emerged from the EBs. SEM images show the crenulation with a normal EB development in WT (**Figure 4E**); a globular-shaped EB was visible in the middle of the leaf crenulation. *KpWUS*, *KdCLV2* and *KpCUC2* AS lines had shorter internodes when compared to WT, resulting in shorter plants (**Figures 4F, K, P**). These AS plants also showed delayed leaf emergence; the youngest developing leaves were barely visible when the previous pair of leaves were already mature (**Figures 4G, L, Q**, arrow). *KpSTM* AS transgenic plants were slightly shorter than WT (**Figure 4U**), but the development of newly emerged leaves was comparable to WT (**Figure 4V**). This short internode phenotype in AS lines was also confirmed by measuring internode length (**Supplementary Figure S1K**). The leaves of *KpWUS*, *KpCUC2* and *KpSTM* AS lines grew slightly wider and shorter in comparison to those in WT (**Figures 4H, R, W**). The leaves in *KpSTM* AS lines were bilaterally asymmetrical (**Figure 4W**). The overall shape of leaves in *KpCLV2* AS lines was similar to WT (**Figure 4M**). AS leaves had fewer leaf crenulations per leaf, except *KpSTM* AS lines, which had a similar number of leaf crenulations to those in WT (**Supplementary Figure S1L**). *KpWUS* AS lines showed severe defects in plantlet formation; detached leaves of *KpWUS* AS lines after 20 days showed no plantlet formation (**Figure 4I**). The crenulations in severe phenotypes of *KpWUS* AS lines showed a cavity without a trace of EBs (**Figure 4J**). After leaf detachment, leaves of *KpCLV2*, *KpCUC2* and *KpSTM* AS lines showed fewer plantlets than observed in WT leaves. Some plantlets emerged but ceased development and died (aborted plantlets, labelled with AP; **Figures 4N, S, X**). SEM images of the leaf crenulations in *KpCLV2* AS lines showed that EB was being formed, however, the structure seemed abnormal (**Figure 4O**); placed in a more exposed position between shallower leaf lobes. Stronger phenotypes in *KpCUC2* AS lines only developed roots without leaf initiation from the EBs (**Figure 4S**) or showed no obvious EB developed (**Figure 4T**). SEM images of the crenulation area in *KpSTM* AS lines further showed phenotypic shallowly positioned EBs (**Figure 4Y**).

### 3.4 Plantlet formation in *KpWUS*, *KpCLV2*, *KpCUC2* and *KpSTM* AS lines

**Figure 5:**
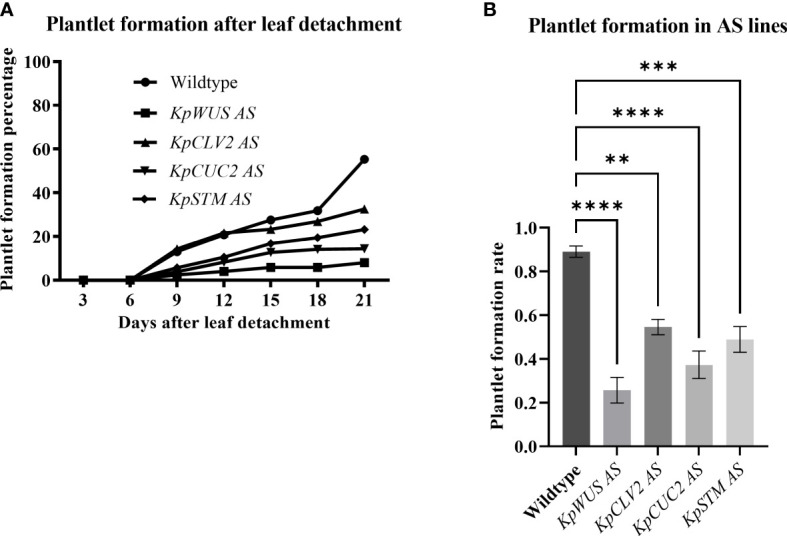
Plantlet formation in antisense (AS) lines. **(A)** Percentage of plantlet formation with time (up to 21 days) after leaf detachment. **(B)** Plantlet formation rate in AS lines after 21 days of leaf detachment. The plantlet formation was scored in 25 leaves of 5 different independent lines. The error bars show SE. The percentages were calculated by dividing the number of crenulations with plantlets by the number of crenulations and then multiplied by 100. The rates were obtained by arcsine-transforming the percentages. One-Way ANOVA with Dunnett’s Multiple Comparison, ns (non-significant); * (P-value <0.01); ** (P-value <0.001); *** (P-value <0.0002) and **** (P-value <0.0001).

To study the role of meristem genes in plantlet formation, we scored plantlet emergence in detached leaves of *K. pinnata* WT and the AS lines. All AS lines showed fewer plantlets compared to WT (**Figure 5A**). We calculated the plantlet formation percentage by dividing the number of crenulations with plantlets by the total number of crenulations. To obtain the plantlet formation rate we arcsine-transformed the percentages to statistically analyse them. The plantlet formation percentage after 21 days of leaf detachment was 55.2% in WT, 8% in *KpWUS* AS lines, 32.5% in *KpCLV2* AS lines, 14.4% in *KpCUC2* AS lines and 23.5% in *KpSTM* AS lines. The plantlets became visible in the crenulations 9 days after leaf detachment in WT and the AS lines. Detached leaves of *KpWUS*, *KpCUC2* and *KpSTM* AS lines formed significantly fewer plantlets than WT between 9 days to 21 days of leaves detachment. Notably, *KpCLV2* AS lines showed fewer plantlet phenotypes after 12 days. All AS lines showed a significantly decreased number of plantlets 21 days after detachment, compared to WT: *KpWUS* AS lines (p=<0.0001), *KpCLV2* AS lines (p=0.0018), *KpCUC2* (p=0.0001) AS lines and *KpSTM* AS lines (p=0.0002) (**Figure 5B**), suggesting that these four meristem genes play a prime role in plantlet formation in the leaves.

**Figure 6:**
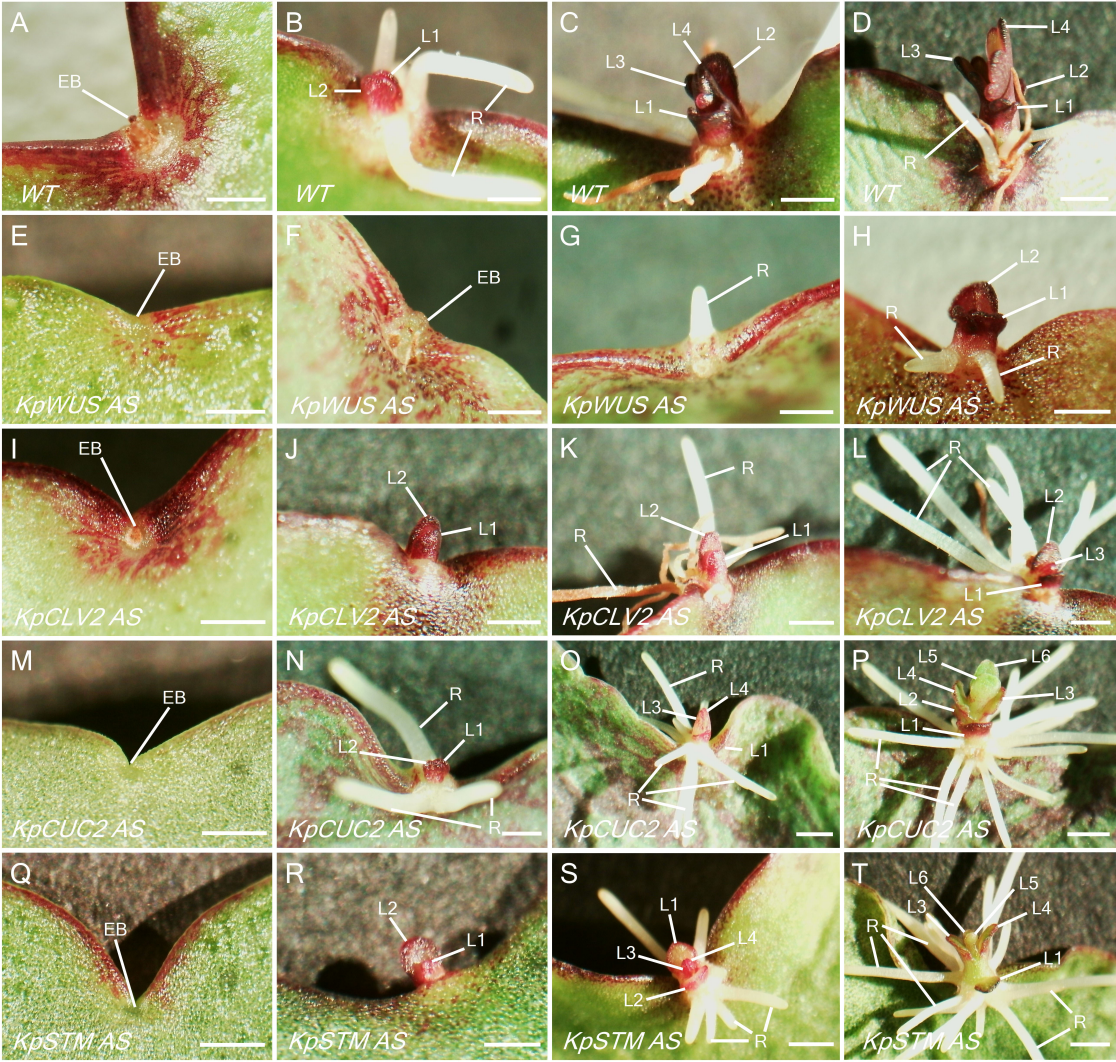
Plantlet phenotypes of *KpWUS*, *KpCLV2*, *KpCUC2* and *KpSTM* antisense (AS) lines. Leaf crenulations in attached leaves **(A, E, I, M, Q)**, after 9 days of leaf detachment **(B, F, J, N, R)**. Plantlet formation after 20 days **(C, G, K, O, S)** and 25 days **(D, H, L, P, T)** of leaf detachment. WT **(A-D)**, *KpWUS* AS lines **(E-H)**, *KpCLV2* AS lines **(I - L)**, *KpCUC2* AS lines **(M-P)** and *KpSTM* AS lines **(Q-T)**. **(C, D)** are the same as **Figures 2C, D**. Scale bar: 1 mm; L1; leaf 1, L2; leaf 2, L3; leaf 3, L4; leaf 4, L5; leaf 5, L6; leaf 6, R; root, EB; epiphyllous bud.

**Figure 7:**
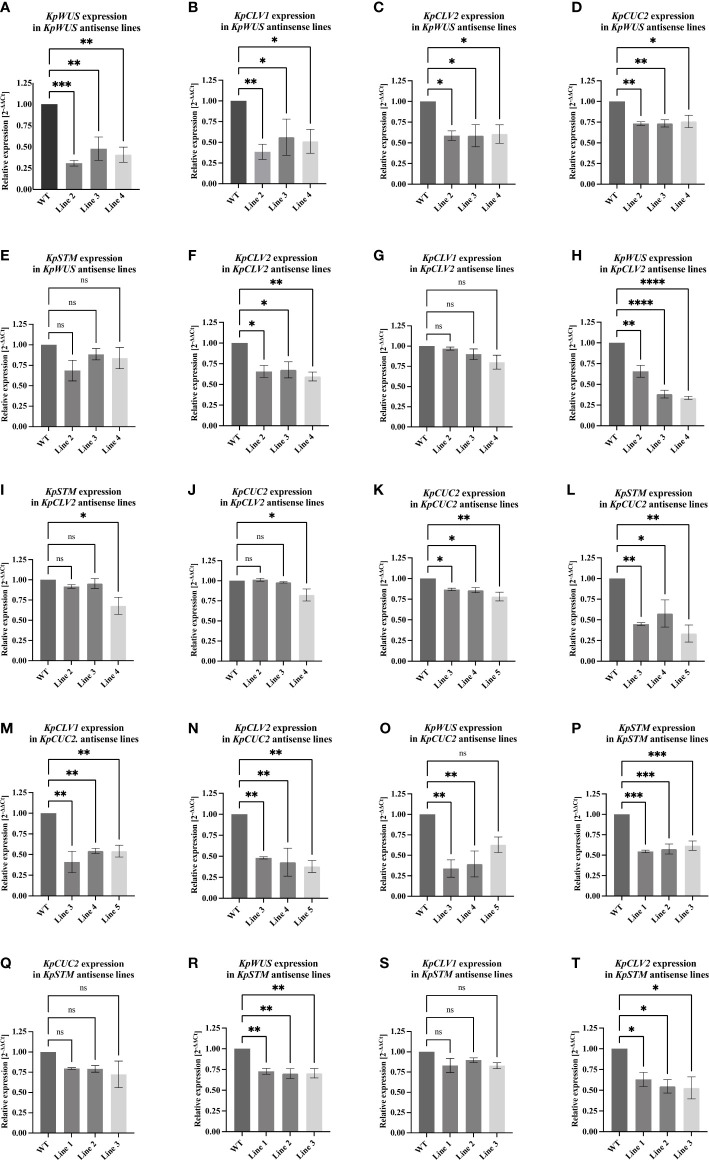
RT-qPCR analysis of *KpWUS*, *KpCLV2*, *KpCUC2* and *KpSTM* antisense (AS) lines. Expression of these genes in *KpWUS* AS lines 2, 3 and 4 **(A-E)**, *KpCLV2* AS lines 2, 3 and 4 **(F-J)**, *KpCUC2* AS lines 3, 4 and 5 **(K-O)** and *KpSTM* AS lines 1, 2 and 3 **(P–T)**. One-Way ANOVA with Dunnett’s Multiple Comparison, ns (non-significant); * (P value <0.01); ** (P value <0.001); *** (P value <0.0002) and **** (P value <0.0001) Errors bars represent the 95% confidence interval.

*K. pinnata* WT mature leaves presented evident EBs in the middle of the leaf crenulations (**Figure 6A**). **Figure 6B** shows developing leaf 1 (L1), L2 and roots 15 days after leaf detachment. Subsequently, L3 and L4 form when L1 and L2 are still growing (**Figure 6C**), however, L3 and L4 seemed to develop faster (**Figure 6D**). The leaves of *KpWUS, KpCUC2 and KpSTM* AS lines showed barely noticeable EBs in mature crenulations when compared to WT leaves (**Figure 6E, M, Q**). Following 15 days of leaf detachment, *KpWUS* AS leaves did not show any visible plantlet formation (**Figure 6F**). **Figure 6G** shows an EB of the *KpWUS* AS lines 20 days after the detachment, with only a small root emergence. Other phenotypes of *KpWUS* AS lines showed small underdeveloped L1 and L2 25 days after leaf detachment (**Figure 6H**). The EBs in *KpCLV2* AS plants were noticeable in mature leaves (**Figure 6I**). L1 and L2 became visible 15 days after leaf detachment, however, no roots emerged and L2 were larger than L1 (**Figure 6J**). **Figure 6K** shows *KpCLV2* AS plantlets 25 days after leaf detachment with developed roots but small asymmetric L1 and L2. Other phenotypes of *KpCLV2* AS plantlets showed several root development but underdeveloped L1 and L2 (**Figure 6L**). 15 days after leaf detachment, *KpCUC2* AS lines presented abundant root development and underdeveloped and phenotypic L1 and L2 (**Figure 6N**). Other phenotypes of *KpCUC2* AS plants also showed abundant root development and slower development of leaf primordia, L1 and L2 remained undeveloped (**Figure 6O**). After 25 days of leaf detachment, some phenotypes of *KpCUC2* AS lines showed abundant root development, underdeveloped L1 and L2, fused L3 and L4 at the base, with no clear delimitation with newly emerged L5 and L6 (**Figure 6P**). After 15 days of leaf detachment, strong phenotypes of *KpSTM* AS lines developed EBs with small asymmetric L1 and L2 (**Figure 7R**). Other AS phenotypes formed numerous roots and phenotypic L1, L2, L3 and L4 emerged symmetrically (**Figure 6S**). Other AS phenotypes of *KpSTM* resembled the *KpCUC2* AS ones, without visible divisions between the developing leaf primordia (**Figure 6T**).

### 3.5 *KpWUS*, *KpCLV1*, *KpCLV2*, *KpCUC2* and *KpSTM* expression in the EBs of *K. pinnata* AS lines

To study the function of key meristem genes and to reveal the genetic means modulating induced plantlet formation in *K. pinnata*, we measured the expression of these genes in the AS lines. RT-qPCR of the crenulate leaf region with EBs of AS *KpWUS* appeared to be significantly downregulated in all *KpWUS* AS lines (**Figure 7A**), confirming that *KpWUS* was successfully downregulated in these AS lines. In the *KpWUS* AS lines, *KpCLV1*, *KpCLV2* and *KpCUC2* were also downregulated (**Figures 7B-D**). However, *KpSTM* expression was similar between the *KpWUS* AS lines and the WT (**Figure 7E**). *KpCLV2* was significantly downregulated in *KpCLV2* AS plants (**Figure 7F**). While *KpCLV1* expression was not affected by *KpCLV2* downregulation (**Figure 7G**), *KpWUS*, *KpSTM* and *KpCUC2* were downregulated in *KpCLV2* AS lines (**Figures 7H-J**). *KpCUC2* AS lines showed downregulation in *KpCUC2*, *KpSTM*, *KpCLV1* and *KpCLV2* (**Figures 7K-N**). *KpWUS* was only downregulated in lines 3 and 4 in comparison to WT (**Figure 7O**). *KpSTM*, *KpWUS* and *KpCLV2* were downregulated in *KpSTM* AS plants (**Figures 7P, R, T**), however, *KpCUC2* and *KpCLV1* expressions were similar to the expression in WT (**Figures 7Q, S**).

### 3.6 *KpWUS* expression in the EBs

**Figure 8:**
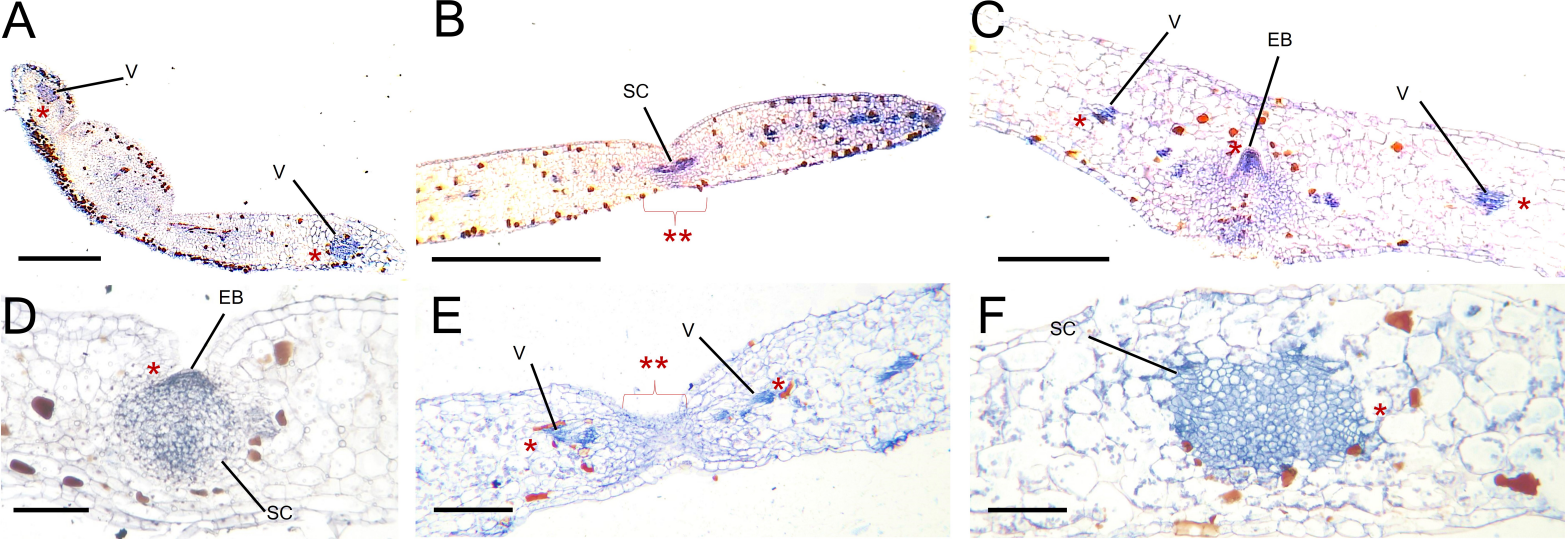
KpWUS signal in epiphyllous buds (EB) of WT, *KpWUS* and *KpCLV2* antisense (AS) lines. *KpWUS* expression in WT; **(A)** newly emerged leaves, **(B)** young leaves <5 cm, **(C)** fully developed EBs, **(D)** EBs 24 hours after leaf detachment, **(E)** in *KpWUS* AS lines and **(F)** in *KpCLV2* AS lines. The KpWUS signal were detected in the vasculature (V) of the leaves and groups of stem cells (SC) in the middle of the crenulations (marked with *). Note no KpWUS signal in the SC in *KpWUS* AS lines (marked with ** with braket) in **(E)** Scale bars: 0.5 mm **(A)**, 1 mm **(B-F)**.

To investigate the location of *KpWUS* expression in the EBs, we performed immunolocalisation using a WUS antibody on the leaf crenulations of WT, *KpWUS* and *KpCLV2* AS plants. *KpWUS* expression was first detected in the main vasculature of WT leaves when crenulations were being formed in the leaf lobes (**Figure 8A**). In growing leaves (< 1 cm long), KpWUS was already detected in a group of small cells in the middle of the leaf crenulation (**Figure 8B**). Mature leaves showed *KpWUS* expression localised to the centre of the developing EB and mildly expressed in the vasculature of leaf lobes, presumably in the parenchyma cells (**Figure 8C**). In fully grown leaves 24 hours after leaf detachment, KpWUS was found to cover a broader expression area including the EBs and the adjacent region (**Figure 8D**). KpWUS signal was not detected in the crenulations of *KpWUS* AS lines24 hours after leaf detachment, but the faint expression in the vasculature of the leaf lobes (**Figure 8E**). In the leaves of *KpCLV2* AS lines, 24 hours after leaf detachment, the KpWUS domain appeared to be broad and un-organised around the leaf crenulation, while no obvious EB structure was observed (**Figure 8F**).

## 4 Discussion

### 4.1 Plantlet formation in *K. pinnata*

The *Kalanchoë* genus evolved to reproduce by forming plantlets on the leaf margins (Allorge-Boiteau, 1996; Garces et al., 2007; Garcěs et al., 2009). Whereas basal *Kalanchoë* species have an obligate sexual reproduction system, more derived inducible plantlet-forming species such as *K. pinnata* can switch from sexual to asexual reproduction by producing plantlets in the leaf crenulations. Previous work reported that the *K. pinnata* EBs stay dormant in the leaves until certain conditions such as leaf detachment and stress induce plantlet formation (Naylor, 1932; Sawhney et al., 1994). Our results confirmed that the EBs in *K. pinnata* are pre-existent in the centre of the leaf crenulations before leaf detachment and stay latent until certain cues trigger plantlet development. It is believed that the disruption of hormone supply to the leaves triggered plantlet formation in *K. pinnata* (Sawhney et al., 1994). Earlier studies performed in *K. pinnata* demonstrated that the plant hormone thidiazuron (TDZ) inhibited plantlet development, whereas ethrel (ETH) promoted plantlet formation (Jaiswal and Sawhney, 2006). It was also revealed that cytokinins zeatin, kinetin, and benzyl aminopurine (BAP) severely disrupted plantlet formation in *K. marnierianum*, an inducible plantlet-forming species (Kulka, 2006).

### 4.2 Differential expression of meristem genes during *K. pinnata* plantlet formation

Molecular studies revealed the expression of *STM* orthologs in the plantlet primordia of *Kalanchoë* plantlet forming species, suggesting their role in plantlet formation in various *Kalanchoë* species (Garces et al., 2007). *STM* in conjunction with several other genes has proven to be essential for the maintenance of stem cells in the SAM and floral meristem in *Arabidopsis* (Endrizzi et al., 1996). This suggests that the meristematic activity, presumably modulated by *STM* during the process of plantlet formation might be also coordinated by other meristem genes. Consistently, our RNA sequencing analysis in the leaf crenulations of *K. pinnata* revealed several meristem genes being expressed in the plantlet primordia. *KpWUS*, *KpWOX11* and *WOX13* being upregulated after leaf detachment, suggesting increasing meristematic activity following the leaf detachment. *WOX* genes have been shown to have essential roles during *Arabidopsis* embryo patterning, organogenesis and stem cell initiation and maintenance (Wu et al., 2007; Ueda et al., 2011; Dolzblasz et al., 2016). Overexpressing lines of *PeWOX11* in *Populus euramericana* (*Pe*) showed an increased number of adventitious root formations (Xu et al., 2015), and *PnWOX11* was found upregulated during root formation in *P. nigra* (Baesso et al., 2020). This suggests that *KpWOX11* might be required for root development in the EBs of *K. pinnata* after leaf detachment. *WOX13* in *Physcomitrella patens* was required for stem cell initiation at the wound margins of detached leaves (Sakakibara et al., 2014). *KpWOX13* might be involved in stem cell establishment and maintenance in the leaf crenulations of *K. pinnata*.

*KpCLV1* and *KpCLV2* were upregulated in the EBs in comparison with the mid-section of the leaf, however, a *s*ignificant downregulation was observed 4 hours after leaf detachment for both genes. In *Arabidopsis*, *WUS* activates *CLV3*, which restricts the *WUS* domain to the OC (Brand, 2000) by binding to the CLV1 ectodomain and the CLV2/CORYNE (CRN) receptor protein complex. Evidence suggests that the CLV1/CLV2 receptor complex interacts with CLV3 to inhibit *WUS* expression (Müller et al., 2008). The downregulation of *KpCLV1* and *2* in the EBs of *K. pinnata* following the leaf detachment might lead to the upregulation of *KpWUS* and other *KpWOX* genes, needed to recruit stem cells for plantlet development.

In the *Kalanchoë* genus, *STM* orthologs were found expressed only in the leaf crenulations of plantlet-forming species (Garces et al., 2007). In *K. pinnata*, *KpSTM* was found slightly upregulated 48 hours after leaf detachment. In *Arabidopsis*, *STM* positively regulates the expression of *CUC1*, *2* and *3* by binding to their promoter (Spinelli et al., 2011). *KpCUC2* and*KpCUC3* were significantly upregulated after leaf detachment. These genes in *Arabidopsis* act redundantly to establish and maintain boundaries around developing organs (Laufs et al., 2004). *CUC2* is also responsible for the presence of leaf serrations in *Arabidopsis* (Kamiuchi et al., 2014). *KpCUC2* and *KpCUC3* might be playing crucial roles in the formation of epiphyllous buds and the process of plantlet formation *via STM*-*CUC* gene pathways.

### 4.3 Phenotypes of meristem gene AS lines

Our results showed that *K. pinnata* AS lines expressing AS fragments of meristem genes (*KpWUS*, *KpCLV2*, *KpCUC2* and *KpSTM*) resulted in no or defective EBs in the leaf crenulations, suggesting that meristem genes are involved in the EBs formation and maintenance. All the AS lines formed significantly fewer plantlets after leaf detachment when compared to WT. Plantlet formation was strongly disrupted in *KpWUS*, *KpCUC2* and *KpSTM* AS lines. Conversely, plantlet formation was less severely affected in the *KpCLV2* AS lines. Evidence suggests that two parallel CLV3 signaling transduction mechanisms restrict *WUS* expression in *Arabidopsis*, either through *CLV1* and *2* heterodimers or *via* independent interaction with *CLV1* or *2*. Only *clv1*-*2* double mutants resulted in severe phenotypes comparable to the ones observed in *CLV3* single mutants (Jeong et al., 1999; Bleckmann et al., 2009). Consistently, our *KpCLV2* AS lines formed fewer plantlets, however, the plantlet formation was not as strongly disrupted as when the expression of other meristem genes was downregulated.

Signals to initiate plantlet formation do not seem to be affected in the AS plants, as the plantlet formation in AS lines also starts on day 9 following the leaf detachment. This suggests that the reduced plantlet formation was mainly due to the defect in the formation of EBs during early leaf development before the detachment, and not due to defects in signaling to trigger plantlet initiation. In support of this, all AS lines showed no or defective EBs in most crenulations (**Figures 6J, O, T, Y**).

Plantlets formed in the AS lines also showed abnormal morphologies. *KpWUS* AS plantlets terminated meristem activity after forming the first leaf pair and small roots, resembling *wus* mutants in *Arabidopsis* (Laux et al., 1996). This suggests that *KpWUS* is important not only for the EB formation but also for the SAM development of plantlets. *KpCUC2* AS plantlets also showed predominant root development and the fusion of plantlet leaf pairs, which reflects the *Arabidopsis cuc2* mutant phenotypes. *CUC2* in *Arabidopsis* delimits the boundaries in the developing organs, and its mutation often generates a fusion of lateral organs (Laufs et al., 2004). Auxin may be involved in plantlet root development in some Kalanchoë species (Kulka, 2008), and in *Arabidopsis*, *CUC2* and the auxin accumulation are mutually exclusive (Bilsborough et al., 2011). Therefore, the root overproliferation phenotypes might be due to an increase of auxin in *KpCUC2* AS plants. *KpSTM* AS plantlets also exhibited root over-proliferation and fused emerging leaves, similar to those of *KpCUC2* AS lines. It has been shown that *STM* and *CUC* genes positively regulate each other in *Arabidopsis* leaf primordia (Spinelli et al., 2011). Similarly, *KpCUC2* might also regulate the expression of *KpSTM* in the EBs of *K. pinnata*. Thus, *KpSTM* AS plants might mimic *KpCUC2* AS phenotypes.

### 4.4 Genetic regulatory relationships among meristem genes

The alteration of expression levels of meristem genes in AS backgrounds revealed insight into regulatory relationships among the genes. *KpWUS* AS lines showed downregulation in *KpCLV1*, *KpCLV2* and *KpCUC2*, suggesting that *KpWUS* positively regulated the expression of these genes in the EBs of *K. pinnata*. Notably, as only the indented regions of the leaf with EBs were harvested for this experiment, it is unlikely that the results could be attributed to the indirect consequences of the decrease of EBs in AS plants. In *Arabidopsis*, the SAM is maintained by the feedback loop between CLV3 and *WUS*. A decrease in *WUS* expression in *Arabidopsis* leads to fewer stem cells, and thus less CLV3 production (Schoof et al., 2000; Somssich et al., 2016). In *Arabidopsis*, *CLV* loss-of-function leads to increased meristematic activity, however, our results indicated that the expression of *KpWUS* was downregulated in the *KpCLV2* attenuated background, suggesting that *KpCLV2* influence the expression of *KpWUS* in the EBs in *K. pinnata*, in a different way as in the SAM of *Arabidopsis*.

Conversely, *KpSTM* was not largely affected by *KpWUS* downregulation; *KpSTM* expression levels showed a trend of decrease but were statistically insignificant. However, *KpWUS* was significantly downregulated in *KpSTM* downregulated background. This suggests that *KpSTM* showed a strong reciprocal regulation to establish the meristematic competency in the EBs of *K. pinnata*, promoting *KpWUS* and subsequently, *KpCLV1* and *2*. Moreover, *KpCUC2* AS lines showed downregulation in *KpWUS*, *KpSTM*, *KpCLV1* and *KpCLV2*. This suggests that *KpCUC2* positively modulates the expression of *KpWUS*, *KpCLV1* and *2 via KpSTM* in the EBs of *K. pinnata.* This suggests the role of *KpCUC2* in the establishment of meristem competency in the leaves of *K. pinnata.* Furthermore, *CUC2* also controls the leaf margin development in *Arabidopsis*, and together with the microRNA (miRNA), *MiR164A* determines the crenulation depth (Nikovics et al., 2006). The leaves of *KpCUC2* AS lines showed shallower leaf crenulations. As the presence of crenulations is a prerequisite for plantlet primordia in *Kalanchoë*, *KpCUC2* is also likely to contribute to plantlet development *via* the formation of crenulations in *K. pinnata*. Notably, *Kalanchoë* species such as *K. marmorata* and *K. eriophylla* that are unable to produce plantlets possess round leaves without leaf crenulations.

Our results showed that key meristem genes were expressed in the leaf crenulations, giving rise to EBs. Furthermore, immunolocalisation revealed the presence of KpWUS in the crenulations of newly forming leaves and a stronger signal at the centre of the EB in the crenulations of mature *K. pinnata* WT leaves. This suggests that meristem activity started early when leaves were being formed from the SAM. In addition, *KpCLV2* AS plants showed a broad and disorganised *KpWUS* domain, resulting in a defective EB arrangement. This evidenced the role of *KpCLV2* in restricting the *KpWUS* domain in the EB. The *CLV*/*WUS* feedback system prevents the over-proliferation of stem cells preserving the SAM size (Yadav et al., 2011). This revealed that the bud maintenance is modulated by the *WUS*-*CLV* complex. Our results also showed that the KpWUS signal increased in the EBs 24 hours following the detachment of the leaves, consistent with our RNA-sequencing analysis. This suggests that *KpWUS* is also involved during plantlet initiation, increasing meristem activity as a response to leaf.

## 5 Conclusion

It appears that *K. pinnata* recruited meristem pathways to facilitate the novel reproductive strategy such as the formation of EBs in the leaf crenulations. Our results demonstrated the ectopic expression of meristem genes in the leaf crenulations of *K. pinnata* assists in plantlet formation as a reproductive process. Downregulation in key meristem genes severely disturbs plantlet formation and defective plantlets. Furthermore, *Kalanchoë* inducible plantlet-forming species represent the first step towards the loss of sexual reproduction within the genus. The importance of *K. pinnata* as a transitional form from sexual to asexual reproduction instigates the acquisition of meristematic competency to achieve the production of plantlets on the leaves.

## Data availability statement

The data presented in the study are deposited in the GenBank repository (https://www.ncbi.nlm.nih.gov/genbank/), the accession numbers are: KpWUS Accession OQ674700, KpCLV2 Accession OQ621804, KpCUC2 Accession OQ621805, KpSTM Accession OQ674701.

## Author contributions

Conceptualization, MK. Methodology, MK and FJ-B. Experiments, FJ-B. Software, FJ-B. Validation, MK and FJ-B. Formal analysis, FJ-B. Writing—original draft preparation, FJ-B. Writing—review and editing, MK and FJ-B. Supervision, MK. All authors contributed to the article and approved the submitted version.

## Supplementary material

Supplementary Figure 1 Genotypes of antisense (AS) lines **(A–H)**. **(I)** showing stem cells (SC) in the meristem of *K. daigremontiana* and negative control without the primary antibody **(J)**. Scale bars: 0.5 mm **(I)** and 1 mm **(J)**. **(K)** Internode length and **(L)** the number of crenulations in wildtype and AS lines. One-Way ANOVA with Dunnett’s Multiple Comparison, ns (non-significant); * (P value <0.01); ** (P value <0.001); *** (P value <0.0002) and **** (P value <0.0001) Errors bars represent the 95% confidence interval.

Supplementary Table 1 Primers for gene cloning and vector assembly.

Supplementary Table 2 List of primers used for genotyping and RT-qPCR.
