# Supplementary material for: Meristem genes are essential for the vegetative reproduction of Kalanchoë pinnata
Source: Front Plant Sci. 2023 May 8;14:1157619. doi: 10.3389/fpls.2023.1157619 (PMC10200927; doi:10.3389/fpls.2023.1157619)
Supplement: Supplementary Table 1 — Primers for gene cloning and vector assembly. [file Table_1.pdf]

Supplementary Tables

Supplementary Table S1 Primers for gene cloning and vector assembly

Module	Insert		Primer 5'→ 3'	Size
p35S	*35S CaMV* promoter	Forw	gtggtctca **GGAG** GCTAGAGCAGCTTGCCAAC	833
		Rev	gtggtctca **CACC** GGTCGATCGACAGATCTGCG	
KpWUS	*K. pinnata WUS* exon 1 (antisense)	Forw	gtggtctct **AAGC**ATGATGGGTGATGACCTTGG	455
		Rev	gtggtctct **GGTG**TTGTTGATGTCTGAGTATTTGG	
KpCLV2	*K. pinnata CLV2* exon 1 (antisense)	Forw	gtggtctct **AAGC**CTCGATGTCAGCAGGAAC	942
		Rev	gtggtctca **GGTG**AATCTCCAGCGATCTACA	
KpCUC2	*K. pinnata CUC2* exon 1 (antisense)	Forw	gtggtctct **AAGC**ACTTGAACAAGTGCGAGCC	258
		Rev	gtggtctca **GGTG**GCATGACCCAGTTGGTCTT	
KpSTM	*K. pinnata STM* exon 1 (antisense)	Forw	gtggtctct **AAGC**CAAATCAACAACTGGTTCATC	333
		Rev	gtggtctct **GGTG**GTAATCTTATTTACATTCATCTAG	
Nos Term	*Nopaline Synthase* Terminus	Forw	gtggtctct **GCTT** GATGATCCCCGATCGTTCAAAC	279
		Rev	gtggtctct **AGCG** GACAGGAGGCCCCGATCTAG	
35S Term	*35S CaMV* Terminus	Forw	gtggtctct **GCTT** GGGACTCTGGGGTTCGGATC	237
		Rev	gtggtctct **AGCG** GGTGATCTGGATTTTAGTACTGG	

Supplementary Table S2 List of primers used for genotyping and RT-qPCR

Target	Primer Name	Sequence 5'→ 3'
M13	*M13* Forw	GTTTTCCCAGTCACGAC
	M13 Rev	CAGGAAACAGCTATGAC
NPTII	*NPTII* Forw	CACAACAGACAATCGGCTGC
	NPTII Rev	GCACGAAGCGGTCAG3
Kd18S	*qKd18S* Forw	AGAAACGGCTACCACATCCAAG
	qKd18S Rev	GACTCATTGAGCCCGGTATTGT

KpWUS	*qKpWUS* Forw	TCATCCACTCCACACATTCACACT
	qKpWUS Rev	CAGCATATCCGAAACCTGACATC
KpCLV2	*qKpCLV2* Forw	ACCTGTCCCACAACTCGCTC
	qKpCLV2 Rev	TTGGCAATGGCGTCG
KpSTM	*qKpSTM* Forw	GCACTACAAATGGCCATACC
	qKpSTM Rev	ATCCTGGAGAGATGTCCATG
KpCUC2	*qKpCUC2* Forw	TCTTCAGCTTGAAAGACCGC
	qKpCUC2 Rev	GTTGGTCTTGTCTCCCTTTG
